# Supplementary material for: Vector competence and immune response of Aedes aegypti for Ebinur Lake virus, a newly classified mosquito-borne orthobunyavirus
Source: PLoS Negl Trop Dis. 2022 Jul 18;16(7):e0010642. doi: 10.1371/journal.pntd.0010642 (PMC9333442; doi:10.1371/journal.pntd.0010642)
Supplement: S1 Table — (DOCX) [file pntd.0010642.s001.docx]

**S1 Table. Primer sequences of genes used for qRT-PCR**

| Gene name | Primers |
| --- | --- |
| EBIV segment S | F：ATGGCATCACCTGGGAAAG  R：TTCCAATGGCAAGTGGATAGAA |
