# Supplementary material for: Vector competence and immune response of Aedes aegypti for Ebinur Lake virus, a newly classified mosquito-borne orthobunyavirus
Source: PLoS Negl Trop Dis. 2022 Jul 18;16(7):e0010642. doi: 10.1371/journal.pntd.0010642 (PMC9333442; doi:10.1371/journal.pntd.0010642)
Supplement: S2 Table — (DOCX) [file pntd.0010642.s002.docx]

**S2 Table. The correlation between EBIV-induced cytopathic effect in BHK-21 cells and CT values of virus RNA by qRT-PCR.**

| **EBIV** | **Virus titer**  **(PFU/mL)** | **virus dose used to inoculate flask (PFU)** | **Days Post-Inoculation that CPE Appeared** | **Ct values** |
| --- | --- | --- | --- | --- |
| No dilution | 1.3 x 10^6^ | 1.3 x 10^5^ | 2 | 17.35 |
| 10^-1^ | 1.3 x 10^5^ | 1.3 x 10^4^ | 2 | 20.7 |
| 10^-2^ | 1.3 x 10^4^ | 1.3 x 10^3^ | 2 | 24.0 |
| 10^-3^ | 1.3 x 10^3^ | 1.3 x 10^2^ | 2 | 27.5 |
| 10^-4^ | 1.3 x 10^2^ | 1.3 x 10^1^ | 3 | 30.6 |
| 10^-5^ | 1.3 x 10^1^ | 1.3 | 4 | 34.6 |
| 10^-6^ | 1.3 | 0.26 | - | 38.0 |
| 10^-6^ | 1.3 | 0.13 | - | 0 |
| 10^-7^ | 0.13 | 0.013 | - | 0 |
