# Supplementary material for: Vector competence and immune response of Aedes aegypti for Ebinur Lake virus, a newly classified mosquito-borne orthobunyavirus
Source: PLoS Negl Trop Dis. 2022 Jul 18;16(7):e0010642. doi: 10.1371/journal.pntd.0010642 (PMC9333442; doi:10.1371/journal.pntd.0010642)
Supplement: Attachment — Submitted filename: Cover Letter and Response to Comments.docx [file pntd.0010642.s005.docx]

**Cover Letter and Response to Comments**

**May 5, 2022**

**Re: Manuscript PNTD-D-22-00238R1**

**Dear Editor(s),**

Attached please find our resubmission of our manuscript “**Vector competence and immune response of *Aedes aegypti* for Ebinur Lake virus, a newly classified mosquito-borne orthobunyavirus**”. We are very grateful for the opportunity to revise our work and very much appreciated the reviewers’ constructive criticisms and suggestions to improve our work.

During the process of revision, we carefully considered each of the criticisms and suggestions and performed experiments as requested. On the following pages, we present our point-by-point responses to each comment. We are confident that our manuscript has become much stronger and are confident that it will now will meet the high expectations of the editorial board for publication in *PLOS Neglected Tropical Diseases*.

Please do not hesitate to contact us in case of any outstanding issue or unclarity.

Best wishes,

*Sincerely,*

Han Xia, Ph.D

Professor

Wuhan Institute of Virology, Chinese Academy of Sciences

Wuhan, 430071, China

Tel: 86-27-87198195;

E-mail: [hanxia@wh.iov.cn](mailto:hanxia@wh.iov.cn)

**Point-by-Point Response**

**General Response**

We would like to express our gratitude to all the reviewers for your expert evaluation of our study. Your comments and critiques have made this revised manuscript much stronger and more focused.

**Specific Response**

**Reviewer's Responses to Questions**

**Methods**

Reviewer #1: -Are the objectives of the study clearly articulated with a clear testable hypothesis stated? Yes

-Is the study design appropriate to address the stated objectives? Yes

-Is the population clearly described and appropriate for the hypothesis being tested? Yes

-Is the sample size sufficient to ensure adequate power to address the hypothesis being tested? Yes

-Were correct statistical analysis used to support conclusions? Yes

**Response:** Thank you.

Reviewer #2: Methods section has a poor english. Should be entirely reviewed. If the objective of the study is additionally to show vector competence, than transmission to a host (animal model) should be included.

**Response:** We apologize for the poor reading experience. The paper has been revised by English Editing Company. We also agree the opinion that it is important to verify whether infected mosquitoes could transmit EBIV to animal modes through biting, which is the limitation of this study. The experiments about the virus transmission to an animal host will be carried on in the future. For this paper, because of the virus RNA positive in saliva, we only indicated the possibility of transmission risk by *Ae. aegypti*. Our methodology on the calculation of virus transmission have also been used in many other researches [1-4].

1. O'Connor O, Calvez E, Inizan C, et al. Vector competence of Aedes aegypti from New Caledonia for the four recent circulating dengue virus serotypes. PLoS Negl Trop Dis. 2020 May;14(5): e0008303.

2. Pocquet N, O'Connor O, Flores HA, et al. Assessment of fitness and vector competence of a New Caledonia wMel Aedes aegypti strain before field-release. PLoS Negl Trop Dis. 2021 Sep;15(9): e0009752.

3. Liu Z, Zhou T, Lai Z, et al. Competence of Aedes aegypti, Ae. albopictus, and Culex quinquefasciatus Mosquitoes as Zika Virus Vectors, China. Emerg Infect Dis. 2017 Jul;23(7):1085-1091.

4. Faizah AN, Kobayashi D, Amoa-Bosompem M, et al. Evaluating the competence of the primary vector, Culex tritaeniorhynchus, and the invasive mosquito species, Aedes japonicus japonicus, in transmitting three Japanese encephalitis virus genotypes. PLoS Negl Trop Dis. 2020 Dec;14(12): e0008986.

Reviewer #3: I have only a few major points for the authors to consider/clarify and a number of minor points that are easy to fix. Please see the "Summary and General Comments" section.

**Response:** Thank you.

--------------------

**Results**

Reviewer #1: -Does the analysis presented match the analysis plan? Yes

-Are the results clearly and completely presented? Yes

-Are the figures (Tables, Images) of sufficient quality for clarity? Yes

**Response:** Thank you.

Reviewer #2: results either needs a extensive review.

**Response:** We have made an extensive review of the results.

Reviewer #3: I have only a few major points for the authors to consider/clarify and a number of minor points that are easy to fix. Please see the "Summary and General Comments" section.

**Response:** Thank you.

--------------------

**Conclusions**

Reviewer #1: -Are the conclusions supported by the data presented? Yes

-Are the limitations of analysis clearly described? No

-Do the authors discuss how these data can be helpful to advance our understanding of the topic under study? Yes

-Is public health relevance addressed? Yes

**Response:** We have added two limitations of analysis in this study, they have been discussed in discussion (Line 402-404 and Line 413-415).

“Further studies on additional species and geographic strains of mosquitoes, such as *Ae. albopictus*, *Cx. quinquefasciatus*, and *Cx. tritaeniorhynchus*, should be conducted to identify the main mosquito vectors for EBIV.”

“However, the issue of whether EBIV-positive mosquitoes are capable of transmitting viruses to naïve vertebrate hosts via biting is yet to be established and further studies on the EBIV-mosquito-host transmission cycle are warranted.”

Reviewer #2: transmission is not an adequate term to use in this manuscript, reasons pointed above. Mainly, no host was exposed to these experimentally infected mosquitoes.

Therefore, I strongly suggest that the term "vector competence" should be changed by "vector capacity" in the manuscript in the current format. Competence should be proved by transmission experiments. Here, the virus was detected to be replicating in mosquito tissues, and viral titers were demonstrated in their saliva, but transmission experiments are lacking.

**Response:** Vector competence is defined as the ability for a vector to acquire and subsequently transmit pathogens, which is most synonymous with transmission efficiency (viral deposition in saliva) in the laboratory settings. Vector capacity is a far more complex parameter which encompasses a marriage between mosquito intrinsic factors (genotype, microbiome, etc.) as well as extrinsic factors, often environmental, that affect behavior, longevity, etc [5]. So, we still used “vector competence” in this manuscript.

5. Azar SR, Weaver SC. Vector Competence: What Has Zika Virus Taught Us? Viruses. 2019 Sep 17;11(9).

Reviewer #3: I have only a few major points for the authors to consider/clarify and a number of minor points that are easy to fix. Please see the "Summary and General Comments" section.

**Response:** Thank you.

--------------------

**Editorial and Data Presentation Modifications**

Reviewer #1: (No Response)

**Response:** Thank you.

Reviewer #2: This manuscript describes the experimental infection of aedes aegypti colonies by oral route and after intrathoracic administration of EBIV, a novel orthobunyavirus discovered on China. It did not report any transmission experiment, so results should be carefully reviewed since mice were not exposed to these experimentally infected mosquitoes. Despite the low viral titers detected in orally infected mosquitoes, the presence of the virus in the ovaries is of biological importance, because it can mean that this virus potentially can be transmitted vertically. To assess this information, male/female prole originated from these experimentally infected mosquitoes should be tested, since more than 20% infection rate is likely to represent this possibility of vertical transmission. Figures have na excelent quality, except for figure 4, which in this format is not possible to read (font size is to small and lacking resolution quality). The most interesting findings however, are the transvcriptome results, since studies evaluating mosquito innate response (related to apoptosis induction, antimicrobial response and ubiquitin pathway) are lacking in literature. Writting of this parto f results, and discussion section, is a better english than the rest of the manuscript.

The entire manuscript needs a english review by a english native speaker, several sentences in the abstract, introduction, material and methods and results, present gramar mistakes (plural/singular mistakes, verb conjugated in the present or future, were it should be presented in the past tense, for instance). Introduction should be more direct and concise to reflect the article content solely. Sentences are in general disconected from each other, they need a conection to let the text more “fluid”, direct, concise. Paragraphs are to big, descriptions should be re-written. Overall, the manuscript should be reviewed to remove non necessary and repetitive descriptions as well.

**Response:** Many thanks! We agree with reviewer. EBIV, as a novel arbovirus, can be the representative of orthobunyaviruses. Similarly, *Ae. aegypti* is widely used as model in mosquito experiments, of which the gene function is also most annotated compared to other common mosquito species. And researches about the interaction between vector and orthobunyaviruses are absent. So, we focus on the interaction between EBIV and mosquitoes in this study, especially the mosquito innate response after infected. There are also several limitations about experiment design presently. The experiments about the virus transmission to a host (animal model) and vertical transmission of EBIV in the mosquito will be carried on in the future.

The Figure 4 has been changed to a higher quality one. We have polished the abstract, introduction, material and methods and results.

**Abstract:**

“Ebinur Lake virus (EBIV) has been verified with highly virulent pathogenic to adult laboratory mice, and antibodies against EBIV have been detected in humans.” – please verify gramar mistakes, redundancy ...(EBIV) has been shown to be highly pathogenic...

**Response:** The sentence has been modified to “Ebinur Lake virus (EBIV), a newly classified orthobunyavirus, is reported to be highly pathogenic in adult mice.” (Lines 25-26).

Does these antibodies were veirified by PRNT, to rule out cross reactions? I sugest to keep it simple and remove this part from abstract (...and antibodies against EBIV have been detected in humans).

**Response:** We have deleted the sentence.

It was showed that EBIV can be transmitted – how? Does the mosquito secrete virus particles in their saliva? How much in viral titers? Viral load? Did the authors performed experimental infection in mice with these experimentally infected mosquitoes? These points should be reflected in the abstract

**Response****:** Thanks for your suggestions. In this study, we have detected EBIV RNA in saliva of EBIV infected mosquitoes through oral feeding, which means the virus particles can cross midgut barrier and disseminate from midgut to mosquitoes’ saliva. According to calculating, the highest viral dose in saliva of single mosquito was 6.3 PFU. We have supplied the information of viral titer in saliva at abstract (Line 31). The experiments about the virus transmission to a host (animal model) will be carried on in the future.

Intrathoracic infection is not the natural infection route for vectors. Since mosquitoes feed on infected blood, oral feeding is the most likely route to reproduce what hapens in nature.

**Response:** We agree with reviewer. For the intrathoracic infection, we would like to detect the changes of gene expression in mosquitoes after they had been infected through this approach. And we also want to confirm the different effect of the barriers (gut and salary gland) for the EBIV transmission.

**Introduction:**

the entire topic should be reformulated to be strait to the point.

**Response:** Many thanks! We have polished introduction.

Line 50: are primarily distributed to three families and one order: Flaviviridae, Togaviridae and Reoviridae belong to orders Amarillovirales, Martellivirales and Reovirales. Sugestion only: use either the four orders and discriminate families, or use the names of the families within Bunyavirales were arboviruses are classified instead.

Please use the current ICTV classification of viral families.

**Response****:** Thanks for your suggestions. The sentence has been modified to “The clinically important MBVs are primarily distributed into four families: *Flaviviridae* [(dengue viruses 1–4 (DENV), Zika virus (ZIKV), West Nile virus, Japanese encephalitis virus], *Togaviridae* (Chikungunya virus), *Reoviridae* (Yunnan orbivirus) and *Peribunyavividae* (Bunyamwera virus)” (line 65-67).

This part of the introduction should be removed:

Lines 56-60: “For example, the most notable flavivirus DENV, the pathogen of dengue 56 fever, has increased dramatically within the past 20 years, and more than 3.9 billion people in 128 countries are reported to be at risk of dengue infection [4]. After ZIKV and WNV Zika virus are introduced into the Western hemisphere, the viruses have a rapid geographical spread, and cause a large number of infections in the population [5,6].”

Introduction should be designed to what is really necessary to understand the importance of the study and the description of novel characteristics of this novel Peribunyaviridae.

**Response:** Thanks for your suggestions. These sentences have been deleted.

Lines 64-66: “So far, the orthobunyaviruses can be discovered in mosquitoes of the different species, such as Ochlerotatus spp., Culex spp. and Aedes spp. [8-10].” Verify gramar mistakes, please: example: “So far, orthobunyaviruses have been descovered to infect different mosquito species...”

**Response:** Corrected.

Line 67: Oropouche virus cause self-limiting acute febrile disease and, in 5% of infected people, mainly children, can cause asseptic meningitis.

**Response:** Thank you. We have added the information in the line 78-79.

Overall, this paragraph seems fragmented, needs a revision to be direct, concise, linked to the rest of the introduction, considerations about flaviviruses, viruses circulating in Europe, should be entirely removed.

It is necessary to explain what is known about this novel virus, and the importance of studying and discovering new viruses before they became a public health problem.

**Response:** We have deleted considerations about flaviviruses, viruses circulating in Europe and added some new introduction about the importance of studying and discovering new viruses.

Observe verb conjugation in this sentence: “Ebinur Lake virus (EBIV), a newly identified orthobunyavirus in China, is isolated from Culex modestus mosquito pools in Xinjiang Province” – replace “is” for “was” or “has been”.

**Response:** Corrected.

Line 87-89: “EBIV can efficiently infect cells derived from rodent, avian, non-human primate, mosquito and human.” Were these tests performed in cell lines or primary cells? Please discriminate in the sentence.

**Response:** We apologize for the unclear description. The sentence has been modified to “EBIV has been shown to efficiently infect cell lines derived from rodent, avian, non-human primate, mosquito and human subjects” (line 87-88).

Lines 89-90: “After the EBIV infection, BALB/c mice show the encephalopathy, hepatic damage and immunological system damages with high mortality” please verify gramar:

Sugestion - EBIV induced encephalopathy, hepatic and immunological system damages with high mortality index in experimentally infected BALB-c mice”

**Response:** Thanks for your suggestion. The sentence has been modified to “Additionally, EBIV induces encephalopathy, hepatic and immunological system damage with a high mortality index in experimentally infected BALB/c mice” (Line 89-90).

Lines 90-91: “Even though there is no report about confirmed human cases of EBIV, the serological proofs for EBIV infection has been detected”

Considering cross-reactions, please indicate wether this study was performed by PRNT or other serological test and introduce this possibility here.

Xia H, Liu R, Zhao L, Sun X, Zheng Z, Atoni E, et al. Characterization of Ebinur Lake Virus

636 and Its Human Seroprevalence at the China-Kazakhstan Border. Front Microbiol. 2019;10:3111: “Of the 211 tested serum samples, 17 were identified as IgM positive (1:4), 26 as IgG positive (1:10), and 4 as both IgM and IgG positive for EBIV by IFA (Supplementary Figure 3). In the participant with fever, higher IgM (10.37% vs. 0) and IgG (14.63 vs. 4.26%) positive rates were observed. Female participants had a much higher IgM-positive rate than male participants (13.95 vs. 4.00%), but no obvious difference was observed for IgG (13.95 vs. 9.6%). Study participants belonging to the >60-year age group had the highest positive rate of both IgM (16.67%) and IgG (12.69%) among all age groups. Furthermore, based on the occupational groups, the proportion of positive samples for IgM and IgG was highest among retirees (20%), followed closely by farmers and factory workers (17.28%). In addition, the neutralizing antibody prevalence of EBIV was 0.95% (2/211) (Table 1). For the two neutralizing antibody-positive cases, one male participant aged 50 years had a 1:8 PRNT90 titer, and the second case was from a female participant aged 50 years with a 1:16 PRNT90 titer. Both of these two study participants were from the Fifth Division of Xinjiang Production and Construction Corps region.”

Does the PRNT was performed only with EBIV? Did the test included other possible circulating orthobunyavirus to compare titers?

**Response:** Yes, the PRNT assay had only been performed with EBIV. A cross-reaction could occur in the IFA or PRNT test, and no other confirmatory data were available to indicate the EBIV can infect humans. We have modified the sentence to “While no confirmed human cases of EBIV have been recorded, IgM and/or IgG positive for EBIV were identified from several serum samples and two neutralizing antibody-positive cases detected via the plaque reduction neutralization test assay in an earlier study by Xia et al.” (line 91-94).

Line 92: Due to the health risk of EBIV: is there any clinical description in humans? If not, remove this sentence.

**Response:** The sentence has been modified to “The findings to date indicate that EBIV presents a potential threat to human and animal health that should be further explored” (line 94-96).

This virus was described in a Culex species. Culex sp. are more frequently associated to orthobunyaviruses than Aedes sp. Why the authors choosed Aedes aegypti instead of Culex mosquitoes colonies to perform this experimental study?

**Response:** According to some researches about vector competence of mosquitoes for orthobunyaviruses, *Ae. aegypti* has been verified to be a potential vector for Bunyamwera virus (BUNV), Cache Valley virus (CVV) and La Crosse virus (LACV) [6-9] (Line 102-103). Therefore, we firstly assess the vector competence of *Ae. aegypti* for EBIV, and vector competence of *Culex* spp. will be carried on in the following experiments. In addition, we tent to study the interaction between EBIV and *Ae. aegypti,* because there are little researches on this and *Ae. aegypti* is a well-established model vector for studies on genes level.

6. Odhiambo C, Venter M, Chepkorir E, Mbaika S, Lutomiah J, Swanepoel R, et al. Vector Competence of Selected Mosquito Species in Kenya for Ngari and Bunyamwera Viruses. Journal of medical entomology. 2014;51(6):1248-53. doi: 10.1603/ME14063.

7. Dutuze MF, Nzayirambaho M, Mores CN, Christofferson RC. A Review of Bunyamwera, Batai, and Ngari Viruses: Understudied Orthobunyaviruses With Potential One Health Implications. Front Vet Sci. 2018;5:69. Epub 2018/05/01. doi: 10.3389/fvets.2018.00069. PubMed PMID: 29707545; PubMed Central PMCID: PMCPMC5906542.

8. Bara JJ, Parker AT, Muturi EJ. Comparative Susceptibility of Ochlerotatus japonicus, Ochlerotatus triseriatus, Aedes albopictus, and Aedes aegypti (Diptera: Culicidae) to La Crosse Virus. Journal of medical entomology. 2016;53(6):1415-21. doi: 10.1093/JME/TJW097.

9. Ayers VB, Huang YS, Lyons AC, Park SL, Dunlop JI, Unlu I, et al. Infection and transmission of Cache Valley virus by Aedes albopictus and Aedes aegypti mosquitoes. Parasit Vectors. 2019;12(1):384. Epub 2019/08/02. doi: 10.1186/s13071-019-3643-0. PubMed PMID: 31366369; PubMed Central PMCID: PMCPMC6670168.

Lines 99-101: whifch is beneficial to better prepare for and respond to potential outbreak of EBIV, and to understand the transmission mechanism of orthobunyavriuses. – I strongly sugest this sentence to be removed, since by the results of previous studies, is not enoguh evidence of human infection. I also recommend that animals from the region should be tested for the virus.

**Response:** Thanks for your suggestion. In the future, we will make the virus detection in the animals from the region. The sentence has been modified to “The collective results provide valuable insights into the interactions between orthobunyavrius and mosquito vector that should aid in assessing the potential risk of EBIV to human health.” (line 107-109).

Material and methods:

Line 111: “The EBIV isolate Cu20-XJ is obtained from Cx. modestus mosquitoes in 2013” – please verify verb conjugation (here is necessary to use past not present time).

**Response:** Corrected.

Line 117: “Eggs of Ae. aegypti (Rockefeller 117 strain) was obtained from Laboratory of...” eggs – plural, was – singular.

**Response:** Corrected.

Lines 120-121: ...”and a relative humidity of 75 ± 5% humidity, approximately.” Humidity is repeated.

**Response:** Corrected.

Lines 125-126: ...”Then put the cups into the mesh cages (30 x 30 x 30 cm), which were kept in the insect incubator with the condition...” ...Cups were kept into the mesh....

**Response:** Corrected.

Lines 132-133: Before oral-infection, five to eight days old, adult mosquitoes were collected by using the mosquito absorbing machine and placed into plastic cups (24oz). And wrap the cup with a cut mosquito net mesh and then cover it with a lid with a hole in the middle.

... “and placed into plastic cups (24oz), wrapped with a mosquito net mesh and covered with a lid containing a oppening in the center”...

**Response:** Corrected.

Line 165: future tense should me avoided in manuscripts. Item 2.4 seems to be a “copy and paste” from Project arquive. Really hard to read.

**Response:** We have corrected the sentence in the line 136. Item 2.4 also has been modified to clearly understand.

Line 211: “Ten mosquitoes as a pool were used to extract total RNA using TRIzol reagent (Invitrogen).” ¬sugestion: “Each pool (n=10 especimens) was subjected to total RNA extraction using...”.

**Response:** Corrected.

Line 226: “The clean reads were performed de novo assembly” did you mean: “Clean reads were used for de novo asembly?”

**Response:** Corrected.

Results:

EBIV can be transmitted by Ae. aegypti through oral feeding – actually, in this manuscript transmission to a vertebrate was not evaluated. The most corrected presentation of this data is as the presence of EBIV titers in aedes aegypti saliva, or that EBIV infect several mosquito tissues.

In order to assess transmission rates, non infected mice should be exposed to these mosquitoes, and their tissues tested for the presence of the virus after the incubation period.

Response: We agree the opinion that it is important to verify whether infected mosquitoes could transmit EBIV to animal modes through biting, which is the limitation of this study. The experiments about the virus transmission to an animal host will be carried on in the future. For this paper, because of the virus RNA positive in saliva, we only indicated the possibility of transmission risk by *Ae. aegypti*. Our methodology on the calculation of virus transmission have also been used in many other researches [1-4].

1. O'Connor O, Calvez E, Inizan C, et al. Vector competence of Aedes aegypti from New Caledonia for the four recent circulating dengue virus serotypes. PLoS Negl Trop Dis. 2020 May;14(5): e0008303.

2. Pocquet N, O'Connor O, Flores HA, et al. Assessment of fitness and vector competence of a New Caledonia wMel Aedes aegypti strain before field-release. PLoS Negl Trop Dis. 2021 Sep;15(9): e0009752.

3. Liu Z, Zhou T, Lai Z, et al. Competence of Aedes aegypti, Ae. albopictus, and Culex quinquefasciatus Mosquitoes as Zika Virus Vectors, China. Emerg Infect Dis. 2017 Jul;23(7):1085-1091.

4. Faizah AN, Kobayashi D, Amoa-Bosompem M, et al. Evaluating the competence of the primary vector, Culex tritaeniorhynchus, and the invasive mosquito species, Aedes japonicus japonicus, in transmitting three Japanese encephalitis virus genotypes. PLoS Negl Trop Dis. 2020 Dec;14(12): e0008986.

271-276: “The mean titers in the EBIV-positive guts at 4 (102.7 PFU ml-1), 7 (102.9 PFU ml-1), and 10 (102.8 PFU 272 ml-1) dpi were higher than those in heads, saliva and ovaries (Fig. 1E). At 14 dpi the viral titer in heads (103.5 PFU ml-1) was similar with the guts (103.2 PFU ml-1) at the same point (Fig. 1F). It was worth noting that the viral titer in saliva could get a highest virus titer at 14 dpi (102.5 PFU ml-1) (Fig. 1G) and at this time point ovaries also get the highest mean virus titer (103.6 PFU ml-1)” – By these titers, is not possible to afirm that aedes aegypti transmit the virus. Is necessary to expose a experimental model to these infected mosquitoes to prove that.

**Response**: Thanks for your suggestion. Mouse infection experiments are important in evaluating the transmission efficiency of vectors, and we will be planned in the future. A previous study by our group showed that > 90% adult BALB/c mice succumbed to death upon intraperitoneal infection with an extremely low dose of EBIV (1-10 PFU), indicating greater pathogenicity of EBIV to mice relative to other orthobunyavirus [10]. The mean amount of virus in the EBIV-positive guts at 4 (100.2 PFU), 7 (158.9 PFU), and 10 (126.2 PFU) dpi were higher than those in heads, saliva and ovaries (Fig. 1E). At 14 dpi the viral titer in heads (632.5 PFU) was similar with the guts (317.0 PFU) at the same point (Fig. 1F). It was worth noting that the viral titer in saliva could get a highest virus titer at 14 dpi (6.3 PFU) (Fig. 1G) and at this time point ovaries also get the highest mean virus titer (796.2 PFU). As a result, the virus may be spread by *Aedes aegypti*.

10. Zhao L, Luo H, Huang D, Yu P, Dong Q, Mwaliko C, et al. Pathogenesis and Immune Response of Ebinur Lake Virus: A Newly Identified Orthobunyavirus That Exhibited Strong Virulence in Mice. Front Microbiol. 2020; 11:625661. Epub 2021/02/19. doi: 10.3389/fmicb.2020.625661. PubMed PMID: 33597934; PubMed Central PMCID: PMCPMC7882632.

Line 297: EBIV was highly susceptible in Ae. aegypti through intrathoracic inoculation – this objective should be more clear. By this title, it was inferred that the virus could not pass by mosquito gut barriers. However, Aedes aegypti was susceptible to virus infection, not otherwise, and by this route of inoculation, viral titers were higher in mosquito tissues than by oral route.

It seems initially that intrathoracic route was used after oral route because viral titers or infection was not that efficient by the most similar route of infection in nature (oral feeding). In other places, it seems that this inoculation was used to evaluate mosquito innate response. Please review the results and abstract section to define these contents more properly.

**Response:** Thanks for your suggestion. The subtitle was modified to “*Ae. aegypti* is highly susceptible to EBIV infection through intrathoracic inoculation” (line 304-305).

“The mean titers in the EBIV-positive guts at 4 dpi (105.0 PFU ml−1) and 10 dpi (105.1 PFU ml−1) were significantly higher than that at 2 dpi (104.1 PFU ml−1, H = 13.70 and p = 0.0033) (Fig. 1E).” – and higher than in orally infected mosquitoes at these time points.

Lines 338-339: “could get very high values” – I do not agree these are very hogh titers. These are medium values representing that the viruses efectively replicated in these tissues.

**Response:** Corrected.

Lines 341-343: “With the increase of days after infection, the transmission rates were also slightly increased, ranging from 34.5-90%. The highest transmission rate...”

I do not think transmission rate is adequate to these results. Secretion/excretion rate, since transmission means actually the infection to be successful in a host exposed to these mosquitoes.

**Response:** Before the experiments starting, we have consulted related literatures and found that transmission rate can be defined as the ratio of the number of mosquitoes with virus-positive saliva to the number of mosquitoes with virus-positive bodies or midguts [11, 12]. So, we used this method and evaluated parameters for the infection efficiency of mosquitoes infected through intrathoracic inoculation (Line 187-192).

11. Jansen S, Heitmann A, Lühken R, Jöst H, Helms M, Vapalahti O, et al. Experimental transmission of Zika virus by Aedes japonicus japonicus from southwestern Germany. Emerg Microbes Infect. 2018;7(1):192. Epub 2018/11/30. doi: 10.1038/s41426-018-0195-x. PubMed PMID: 30482893; PubMed Central PMCID: PMCPMC6258727.

12. Souza-Neto JA, Powell JR, Bonizzoni M. Aedes aegypti vector competence studies: A review. Infection, genetics and evolution: journal of molecular epidemiology and evolutionary genetics in infectious diseases. 2019;67:191-209. Epub 2018/11/23. doi: 10.1016/j.meegid.2018.11.009. PubMed PMID: 30465912; PubMed Central PMCID: PMCPMC8135908.

Discussion: overall, this is the most well written parto f the manuscript. Results are properly explored. I would like to know what the authors have to say about Ae. Aegypti been just a model, as the natural vector or reservoir might be a Culex species. Also, if the authors intend to perform mosquito experimental infection and test their transmission potential to experimental models (mice). Oral infection is the natural route of vector infection. Intrathoracic inoculation is not a natural route of infection. What is the explanation for the discrepant results between these two routes?

**Response:** According to some researches about vector competence of mosquitoes for orthobunyaviruses, *Ae. aegypti* has been verified to be a potential vector for Bunyamwera virus (BUNV), Cache Valley virus (CVV) and La Crosse virus (LACV) [6-9] (Line 102-103). Therefore, we firstly assess the vector competence of *Ae. aegypti* for EBIV, and vector competence of *Culex* spp. will be carried on in the following experiments.

The explanation of the discrepant results between these two routes has been put in line 420-424 as below:

“Following intrathoracic injection, EBIV bypassed the midgut barrier and was able to rapidly infect the whole mosquito body, including saliva and ovary (Fig. 2G to H). The distinct results obtained with the two routes indicate that the midgut of *Ae. aegypti* is the main barrier to EBIV transmission.”.

6. Odhiambo C, Venter M, Chepkorir E, Mbaika S, Lutomiah J, Swanepoel R, et al. Vector Competence of Selected Mosquito Species in Kenya for Ngari and Bunyamwera Viruses. Journal of medical entomology. 2014;51(6):1248-53. doi: 10.1603/ME14063.

7. Dutuze MF, Nzayirambaho M, Mores CN, Christofferson RC. A Review of Bunyamwera, Batai, and Ngari Viruses: Understudied Orthobunyaviruses With Potential One Health Implications. Front Vet Sci. 2018;5:69. Epub 2018/05/01. doi: 10.3389/fvets.2018.00069. PubMed PMID: 29707545; PubMed Central PMCID: PMCPMC5906542.

8. Bara JJ, Parker AT, Muturi EJ. Comparative Susceptibility of Ochlerotatus japonicus, Ochlerotatus triseriatus, Aedes albopictus, and Aedes aegypti (Diptera: Culicidae) to La Crosse Virus. Journal of medical entomology. 2016;53(6):1415-21. doi: 10.1093/JME/TJW097.

9. Ayers VB, Huang YS, Lyons AC, Park SL, Dunlop JI, Unlu I, et al. Infection and transmission of Cache Valley virus by Aedes albopictus and Aedes aegypti mosquitoes. Parasit Vectors. 2019;12(1):384. Epub 2019/08/02. doi: 10.1186/s13071-019-3643-0. PubMed PMID: 31366369; PubMed Central PMCID: PMCPMC6670168.

Reviewer #3: I have only a few major points for the authors to consider/clarify and a number of minor points that are easy to fix. Please see the "Summary and General Comments" section.

**Response:** Thank you.

--------------------

**Summary and General Comments**

Reviewer #1: Line 47 - Mosquito-borne viruses (MBVs), as a group of heterogeneous RNA viruses, naturally survive in both mosquitoes and vertebrate hosts, and are the aetiological agents of many human diseases.

"Mosquito-borne viruses" may be confusing because of the insect-specific viruses that do not replicate in vertebrate cells. It seems that Ebinur Lake virus has demonstrated capacity to infect vertebrate and invertebrate cell lines so it can be categorized as an arbovirus, which is a term well-established. Also, viruses replicate rather than survive.

I suggest changing to:

Arthopod-borne viruses (arbovirus), as a group of heterogeneous RNA viruses, naturally replicate in both mosquitoes and vertebrate hosts, and are the aetiological agents of many human diseases.

**Response:** Thanks for your suggestions. Generally, Mosquito-borne viruses (MBVs), also known as moboviruses, are associated mainly with mosquitoes and able to infect humans and other vertebrates [13]. And Mosquito-specific viruses are defined as the insect-specific viruses [14]. For this, we still use the “Mosquito-borne viruses (MBVs)”. And the “survive” have been changed into “replicate”.

13. Schmidt-Chanasit J, Agboli E, Jost H. Special Issue "Mosquito-Borne Virus Ecology". Viruses. 2022;14(2). Epub 2022/02/27. doi: 10.3390/v14020357. PubMed PMID: 35215949; PubMed Central PMCID: PMCPMC8875898.

14. Agboli E, Leggewie M, Altinli M, Schnettler E. Mosquito-Specific Viruses-Transmission and Interaction. Viruses. 2019;11(9). Epub 2019/09/20. doi: 10.3390/v11090873. PubMed PMID: 31533367; PubMed Central PMCID: PMCPMC6784079.

Line 49 - The medically important MBVs are primarily distributed to three families and one order: the Flaviviridae family [dengue viruses 1-4 (DENV), Zika virus (ZIKV), West Nile virus (WNV), Japanese encephalitis virus (JEV), etc], the Togaviridae family [Chikungunya virus (CHIKV), etc], the Reoviridae order [(Banna virus (BAV), etc) and Bunyavirales order [Rift Valley fever virus (RVFV), etc] [2, 3].

I suggest changing to:

Line 49 - The medically important arboviruses are primarily distributed to four families: Flaviviridae [dengue viruses 1-4 (DENV), Zika virus (ZIKV), West Nile virus (WNV), Japanese encephalitis virus (JEV), etc], Togaviridae [Chikungunya virus (CHIKV), Mayaro virus (MAYV) etc], Reoviridae [Banna virus (BAV), etc], and Peribunyaviridae [Oropouche orthobunyavirus (OROV) etc] [2, 3].

**Response:** Thanks for your suggestions. We have corrected that.

Line 57 - After ZIKV and WNV Zika virus are introduced into the Western hemisphere, the viruses have a rapid geographical spread, and cause a large number of infections in the population

I suggest changing to:

After ZIKV and WNV were detected in the Western hemisphere, they rapidly spread causing large number of neurological disorders mainly in Brazil and United States, respectively.

**Response:** Thanks for your suggestions. According to the opinion of reviewer 2, the sentences has been deleted.

Line 60 - change "mosquito-borne" by "arbovirus", and add the proper references.

**Response:** Thanks for your suggestions. Generally, Mosquito-borne viruses (MBVs), also known as moboviruses, are associated mainly with mosquitoes and able to infect humans and other vertebrates [13]. And Mosquito-specific viruses are defined as the insect-specific viruses [14]. For this, we still use the “Mosquito-borne viruses (MBVs)” and don’t need to add the other references.

13. Schmidt-Chanasit J, Agboli E, Jost H. Special Issue "Mosquito-Borne Virus Ecology". Viruses. 2022;14(2). Epub 2022/02/27. doi: 10.3390/v14020357. PubMed PMID: 35215949; PubMed Central PMCID: PMCPMC8875898.

14. Agboli E, Leggewie M, Altinli M, Schnettler E. Mosquito-Specific Viruses-Transmission and Interaction. Viruses. 2019;11(9). Epub 2019/09/20. doi: 10.3390/v11090873. PubMed PMID: 31533367; PubMed Central PMCID: PMCPMC6784079.

Line 65 - change "Ochlerotatus spp., Culex spp. and Aedes spp. [8-10]" by "Ochlerotatus spp., Culex spp. and Aedes spp. and also midges, as Culicoides paraensis, which is a vector of OROV in South America [8-10]"

**Response:** Thanks for your suggestions. The sentences have been modified to “So far, orthobunyaviruses have been detected in mosquitoes of different species, including *Ochlerotatus* spp., *Culex* spp. and *Aedes* spp., and midges, such as *Culicoides* paraenesis, a vector of Oropouche virus (OROV) in South America” (line 73-76).

From line 92 to 95 - Authors should indicate here why not using Culex spp for the experimental infection, or at least describe if any experimental work has been done with Culex spp. EBIV was detected in Culex modestus, so it would make sense evaluate the vector capacity and competence of the most common Culex spp. in China as well. This topic also needs to be discussed in the discussion section.

**Response:** According to some researches about vector competence of mosquitoes for orthobunyaviruses, we found that *Ae. aegypti* can be a potential vector for Bunyamwera virus (BUNV), La Crosse virus (LACV) and Cache Valley virus (CVV) [6-9] (Line 102-103). Therefore, we firstly chose *Ae. aegypti*, and vector competence of *Culex* spp. will be carried on in the following experiments. We have added these sentences “This study explored the vector competence of *Ae. aegypti* for EBIV in view of the susceptibility of this mosquito species to other orthobunyaviruses [22-24, 61]. For instance, *Cx. quinquefasciatus* is reported to be refractory to BUNV, but not *Ae. aegypti* [21]. Further studies on additional species and geographic strains of mosquitoes, such as *Ae. albopictus*, *Cx. quinquefasciatus*, and *Cx. tritaeniorhynchus*, should be conducted to identify the main mosquito vectors for EBIV.” (line 399-404) to the discussion section.

6. Odhiambo C, Venter M, Chepkorir E, Mbaika S, Lutomiah J, Swanepoel R, et al. Vector Competence of Selected Mosquito Species in Kenya for Ngari and Bunyamwera Viruses. Journal of medical entomology. 2014;51(6):1248-53. doi: 10.1603/ME14063.

7. Dutuze MF, Nzayirambaho M, Mores CN, Christofferson RC. A Review of Bunyamwera, Batai, and Ngari Viruses: Understudied Orthobunyaviruses With Potential One Health Implications. Front Vet Sci. 2018;5:69. Epub 2018/05/01. doi: 10.3389/fvets.2018.00069. PubMed PMID: 29707545; PubMed Central PMCID: PMCPMC5906542.

8. Bara JJ, Parker AT, Muturi EJ. Comparative Susceptibility of Ochlerotatus japonicus, Ochlerotatus triseriatus, Aedes albopictus, and Aedes aegypti (Diptera: Culicidae) to La Crosse Virus. Journal of medical entomology. 2016;53(6):1415-21. doi: 10.1093/JME/TJW097.

9. Ayers VB, Huang YS, Lyons AC, Park SL, Dunlop JI, Unlu I, et al. Infection and transmission of Cache Valley virus by Aedes albopictus and Aedes aegypti mosquitoes. Parasit Vectors. 2019;12(1):384. Epub 2019/08/02. doi: 10.1186/s13071-019-3643-0. PubMed PMID: 31366369; PubMed Central PMCID: PMCPMC6670168.

Line 117 - Authors need to explain and discuss at some point in the manuscript the advantages and disadvantages of using the Rockefeller strain of Ae. aegypti for these experiments. We know that different populations of Ae. aegypti can have different vectorial capacity for other arboviruses, so it would be helpful for the readers to know why not using a wild population of Ae. aegypti reared from eggs collected in the field in China. Wild populations would reflect a more realistic vectorial capacity due ecological and biological characteristics that could interfere with EBIV replication, as the infection by insect-specific viruses etc. Perhaps this can be considered a first step for the vector competence analysis, but it needs be clear for the reader the reasons for the chosen approach.

**Response:** Thanks for your suggestions. The Rockefeller strain of *Ae. aegypti* has been commonly used to assess ZIKV, CHIKV, West Nile virus (WNV) and DENV [12]. For this, in this manuscript we choose this strain to study the infection of EBIV on *Aedes* mosquitoes. In the future, the different populations of *Ae. aegypti* will be used to assess the vector competence for EBIV. This study is subject to several limitations, one is comparing vector competence of different mosquito species for EBIV is not designed in this research. The other is whether EBIV-positive mosquitoes are capable of transmitting viruses to naïve vertebrate hosts is not detected currently, so further studies are needed to conduct and complete the researches of EBIV-mosquito-host cycle. While we tent to study the interaction between EBIV and *Ae. aegypti,* because there are little researches on this and *Ae. aegypti* is a well-established model vector for studies on genes level.

12. Souza-Neto JA, Powell JR, Bonizzoni M. Aedes aegypti vector competence studies: A review. Infection, genetics and evolution: journal of molecular epidemiology and evolutionary genetics in infectious diseases. 2019; 67:191-209. Epub 2018/11/23. doi: 10.1016/j.meegid.2018.11.009. PubMed PMID: 30465912; PubMed Central PMCID: PMCPMC8135908.

Line 136 - Mosquitoes were fed with supernatant of EBIV-infected BHK cells

Line 143 - Under the dissecting microscope, insert the loaded needle (filled with supernatant of EBIV-infected BHK cells)

I noticed the virus concentration used for inoculation and blood meal is described in the results section, but I wonder if it would not be easier for the reader having that information here at the material and methods.

**Response:** Sorry for the inconvenience. The different concentrations of viruses have been listed at item 2.3 in material and methods.

Line 147 - "nl of virus" My understanding is that authors did not concentrate virus for inoculation, so here "virus" needs to be replaced by supernatant of EBIV-infected BHK cells.

**Response:** Corrected.

Line 186 - "mice anti-EBIV-NP" Was this primary antibody prepared by the authors in previous study? If so, cite here. Is this mouse hyper immune ascitic fluid? If so, provide that information here.

**Response:** The antibody used in this paper is made in lab previous study, but there is not a paper described the preparation process. The antibody is derived from mice serum and the information has been added in Line 218.

Line 248 to 252 could be transferred to the material and methods section.

**Response:** Thanks for your suggestions. We have transferred those to line 144-149.

Line 252 to 256 - It is not very clear if titration was done by the equation described on line 177 and 178 or plaque assay. If by the described equation, please explain it better, citing references that support the calculation used. If plaque assay was used, describe the method at some point at material and methods.

**Response:** Titration was done by the equation described on Line 208. And reference has been cited in Line 210.

Line 381-384 - "we compared the differences of gene expression between the intrathoracic inoculation and mock-injected mosquitoes at the same time point". Please, make clear here, or later in line 470-473 in the discussion section, the reason for not using orally-infected mosquitoes to evaluate down-regulation of immune-related genes.

**Response:** Through intrathoracic inoculation, the mosquitoes can be completely infected by EBIV, it is easy for us to find the related genes with the virus infection.

Lines 468-469 and 482-484 describe the same potential for vertical transmission.

**Response:** We have deleted the sentence in lines 468-469.

Discussion section - Overall, I believe the discussion section can be improved. I missed in the discussion section the limitations of this study, which could enrich the discussion of this laborious study. Authors could discuss the potential different responses using different field Ae. aegypti populations, the experimental infection with Culex spp, etc. Also, the increasing followed by reduction in dissemination and infection rates observed at 10 dpi etc. Discussion of down-regulation of genes, for instance, is restricted to a couple of lines and should be better explored.

**Response:** Thanks for your suggestions. The discussion of limitations of this study and the potential different responses using different field *Ae. aegypti* populations have been added in Lines 402-404 and Lines 413-415.

Reviewer #2: Overall, the main goal of this study is to demonstrate that viral replication alters gene expression in an experimental vector model. Therefore, after an extensive review, i recommend this article to be evaluated again by reviewers.

**Response:** Thanks for your suggestions. The transcriptional response of *Ae. aegypti* to EBIV infection is to explain the potential mechanism about EBIV infection in mosquito. We have completed the discussion of innate response after infected.

Reviewer #3: This manuscript describes the EBIV- Aedes aegypti interactions at the vector competence level. Two infection ways possess divergent infection, dissemination rate, and ovary infection rates at different dpi. The authors also present the morphology of EBIV in different organs via IFA and EM. Finally, the transcriptome data provide the relevant immune/ metabolism genes expression profile, potentially associated with the vector transmission capability. The work is well-executed, with an adequate number of replicates and controls. The study has generated novel results of interest and increased our knowledge on this newly classified mosquito-borne orthobunyavirus and the vector. I have only a few major points for the authors to consider/clarify and a number of minor points that are easy to fix.

1-I wonder if the title should be more specific to better reflect the results obtained in this work.

**Response:** Thanks for your suggestions. In my opinion, the title has been modified to “Vector competence and immune response of *Aedes aegypti* for Ebinur Lake virus, a newly classified mosquito-borne orthobunyavirus”

2- The English needs to be clarified so that the reader clearly understands what is being said. This must be done throughout the document, but here are some examples:

L21. ... diseases has been increasing in the last...

L25. ... it is necessary to assess mosquitoes' vector capacity for EBIV to predict its risk to...

L27... was shown that EBIV could be...

L33. Delete “all”

L33. …demonstrated EBIV could alter…

L34. processes

**Response:** We apologize for the grammar mistakes. This paper has been revised by English Editing Company.

3-I have some comments on the methods. The authors need to be clarified.

a. L112, please provide the information for mice, strain, age.

b. L114, a reference, needs to cite to describe this plaque assay.

c. L113, adult female?

d. It would be better to provide the numbers of mosquitoes in each experiment, such as viral detection, IFC, and EM.

e. a reference need to cite for the rates used in this manuscript.

f. filter information for confocal.

**Response:**

a. This sentence has been deleted.

b. The reference about plaque assay has been cited in Line 119.

c. “mosquito” is changed into “adult female mosquito”.

d. The numbers of mosquitoes in viral detection was more than 30 mosquitoes per group, IFA was 30/15 per group, EM was 20 per group. The specific number was added in Line 141, Line 174, Line 213, Line 214 and Line 232, respectively.

e. The reference has been cited in Line 167.

f. Corrected.

4- I have the feeling that this 3.7 of the Results section is some kind of last minute add-on. I would suggest adding more words in the relevant pathways, especially for the functions in the modulation of virus infection.

**Response:** Thanks for your suggestions. We have added more words to describe the relevant pathways as follow:

“Toll and Imd signaling pathways are implicated in the protective response of mosquito to EBIV infection [27]. Endocytosis plays an important role in host entry of several virus types, such as flaviviruses and bunyaviruses [39, 40].” (line 464-467).

5- I favor future work, and possible points would be investigated in the Discussion section, but some words in the discussion need to be said for the transcriptome data. I have some suggestions for the authors to consider. For example, the function of the up-regulation genes in immune response; how the metabolism pathways work on the virus infection?

**Response:** Thanks for your suggestions. We have added some discussion about that in the discussion (line 464-477).

--------------------
