# Supplementary material for: Vector competence and immune response of Aedes aegypti for Ebinur Lake virus, a newly classified mosquito-borne orthobunyavirus
Source: PLoS Negl Trop Dis. 2022 Jul 18;16(7):e0010642. doi: 10.1371/journal.pntd.0010642 (PMC9333442; doi:10.1371/journal.pntd.0010642)
Supplement: Attachment — Submitted filename: Response to Comments.docx [file pntd.0010642.s006.docx]

**Point-by-Point Response**

**Methods**

Reviewer #2: objectives are clearly stated, study design seems to be firstly made to prove oral natural infection occur in this species and virus reach salivary glands. In a second moment, the authors choosed intrathoracic inoculation to overcome gut barrier of virus dissemination to stablish persistent salivary gland infection and study which genes are modulated by virus replication in mosquitoes (Ae aegypti). I am not convinced by the results that Ae aegypti is a good choice, either that this species could sustain virus transmission of this virus.

sample size is ok

ethical or regulatory requirements are ok too.

**Response:** Thank you. Further studies on additional species and geographic strains of mosquitoes, such as *Aedes albopictus*, *Culex quinquefasciatus*, and *Culex tritaeniorhynchus*, will be conducted to identify the main mosquito vectors for EBIV. In the future, the experiments about the virus transmission to a host (animal model) will be carried on.

Reviewer #3: (No Response)

--------------------

**Results**

Reviewer #2: Current version is a lot more clear and in a better fashion.

-The analysis presented match the analysis plan? Abstract contains few affirmations not sustained by the presented results that should be reviewed again. Ovary infection rates and saliva titers after intrathoracic infection, and gene expression modulation by viral infection are important results of the study.

-Are the results clearly and completely presented? See comments above.

-Are the figures (Tables, Images) of sufficient quality for clarity? Yes. Figures are very clear.

**Response:** Thank you. We have modified the depiction of abstract (Line 26-33, Line 36-37, Line 38-43 and Line 46-47).

Reviewer #3: (No Response)

--------------------

**Conclusions**

Reviewer #2: -Are the conclusions supported by the data presented? See considerations about abstract. Discussion is a lot improved.

-Are the limitations of analysis clearly described? It is a lot improved in this version.

-Do the authors discuss how these data can be helpful to advance our understanding of the topic under study? Yes.

-Is public health relevance addressed? These studies do not implicate public health directly. Few data relate this pathogen to humans in literature. This study contributes to understand Ae aegypti replication of the virus. Main findings are related to mosquito model, mosquito immune response activation, importance of gut barrier to limit virus dissemination in mosquito body to reach salivary glands, possible mosquito vertical transmission since the virus reached ovaries. Biological importance in an anthropophilic mosquito species is well demonstrated.

**Response:** Thanks for your suggestion. We have modified the depiction of author summary (line 52-53, Line 55-58 and Line 62-70).

--------------------

**Summary and General Comments**

Reviewer #2: Main consideration:

This version of manuscript is a lot more uniform, direct and concise. Importance of invertebrate model studies is clearly demonstrated by the findings, since EBIV infected ovary tissues (reflecting it possibly is transmitted to offsprings) and viral titers are present in saliva. Also, demonstrate gut barrier limited virus dissemination after natural infection and, virus replication leaded to gene expression related to mosquito immune response. These findings are well demonstrated and should be evidenced in the abstract. Therefore, abstract should be reformulated to properly define results and findings of the study.

Public health importance should be minimized since i. human transmission was not accessed in this manuscript; ii. the study did not show a high frequency of positivity in the saliva of Ae aegypti when infection is made with high viral titer through oral route, only when non-natural, intrathoracic route is used. Gut barrier probably limited the virus dissemination to salivary glands, leading to few mosquitoes with virus in their saliva after oral infection, when considering the total number of mosquitoes included in the experiment/evaluated in each time period (figure 1G). Therefore, this observations should be tacking into consideration to complete review of the manuscript.

By the results, is not possible to say this species could spread the virus, since a high number of mosquitoes infected through oral route did not show virus titers in saliva.

Should reflect these observations:

“Following intrathoracic injection, EBIV bypassed the midgut barrier and was able to rapidly infect the whole mosquito body, including saliva and ovary (Fig. 2G to H). The distinct results obtained with the two routes indicate that the midgut of Ae. aegypti is the main barrier to EBIV transmission.”

In my opinion, oral infection does not seem to be very efficient in this species of mosquito, next studies should consider to use other mosquito population.

Seems gut barrier limits the establishment of persistent infection in salivary glands of Ae aegypti even when a high viral load is inoculated. Only a few mosquitoes of this species are infected through natural vector infection route, considering the number of mosquitoes included in the experiment.

Therefore, implications to public health at this point, should not be made.

Author summary is a lot better focused in the biological importance of the main findings of the study. Abstract should be reformulated to continuous that line of thinking.

Lines 94-95: “it poses a considerable risk to public health and may offer a new threat to human or animal health and economic prosperity” –is not enough data to affirm this virus is a public health threat. In my opinion, the study should exclude these affirmations and reflect the important findings and conclusions related to mosquitoes. As far as is possible to comprehend, this virus is important to animal health, not yet proved with sufficient data that it can cause an outbreak involving humans.

Discussion section should be reflected on abstract writing.

**Response:** Thanks for your suggestion. We have deleted the sentence in line 67-70. We have modified the abstract. The paper also downplays the relevance of Public health. Line 453: “poses a significant risk to public health and” has been deleted; Line 484-485 has been revised to “which indicates that with increasing viral titers in host blood, the risk of EBIV transmission to vertebrates is bigger.”

Reviewer #3: (No Response)
